# The small molecule inhibitor SU056-mediated targeting of YB1 inhibits the Rb pathway in triple negative breast cancer tumors

**DOI:** 10.64898/2026.09.05.749600

**Published:** 2026-09-10

**Authors:** Wei Wang, Yeaji Kim, Xiaotong Zhao, Kristen Weber Bonk, Ruth Keri, Bin Su, Khalid Sossey-Alaoui

## Abstract

**Background:**

Triple-negative breast cancer (TNBC) tumors lack expression of estrogen receptor (ER), progesterone receptor (PR), and HER2, limiting the availability of targeted therapeutic options. As a result, TNBC is characterized by a high propensity for metastasis, rapid recurrence, and poor overall prognosis. Despite significant research efforts, the molecular mechanisms that drive TNBC progression and metastasis remain incompletely understood.

Y-box binding protein-1 (YB1) is a multifunctional DNA/RNA-binding protein that regulates transcription, translation, and mRNA stability. Aberrant activation of YB1 has been implicated in multiple oncogenic processes, including proliferation, survival, invasion, metastasis, and therapy resistance in several cancer types, including TNBC. Our investigations have revealed a central role for YB1 in regulating key hallmarks of cancer that drive TNBC tumor progression and metastatic dissemination.

**Methods:**

Cell viability and drug sensitivity in TNBC cell lines were assessed using the MTT assay to determine IC₅₀ values for pharmacologic inhibitors. Western blotting and quantitative RT-PCR were used to quantify protein and mRNA expression levels in TNBC cell lines and tumor samples. Oncogenic phenotypes were evaluated using 2D colony formation assays, 3D tumorsphere growth assays, limiting dilution, and transwell migration assays to assess proliferative, stem-like, and migratory capacities. Cell cycle progression was analyzed using flow cytometry–based assays. In vivo therapeutic efficacy was evaluated using preclinical xenograft and patient-derived xenograft (PDX) mouse models of TNBC to assess tumor growth and metastatic progression following treatment.

**Results:**

Our studies demonstrate that the small-molecule inhibitor SU056, which specifically targets YB1 for degradation, significantly suppresses the oncogenic behavior of TNBC cell lines and tumors. Pharmacologic inhibition of YB1 reduced TNBC cell proliferation, clonogenic growth, tumorsphere formation, stemness and migratory capacity. Importantly, combined treatment with SU056 and the CDK4/6 inhibitor palbociclib enhanced the inhibitory effect on TNBC cell growth and tumor progression compared with either agent alone. Mechanistically, both genetic and pharmacologic targeting of YB1 suppressed TNBC tumor growth and metastatic potential through modulation of the Cyclin D/CDK4/6/Rb signaling axis, leading to inhibition of cell cycle progression and induction of G1 arrest.

**Conclusion:**

Collectively, these findings identify SU056/CDK4/6i combination as a potential therapeutic option for the treatment of Rb ^+^ -TNBC.

## Full Text Availability

The license terms selected by the author(s) for this preprint version do not permit archiving in PMC. The full text is available from the preprint server.

